# Supplementary material for: New Insights in blaKPC Gene Mobilization in Pseudomonas aeruginosa: Acquisition of blaKPC-3 and Identification of a New Tn2-like NTE Mobilizing blaKPC-2
Source: Antibiotics (Basel). 2025 Sep 19;14(9):947. doi: 10.3390/antibiotics14090947 (PMC12466754; doi:10.3390/antibiotics14090947)
Supplement: Supplementary file 1 [file antibiotics-14-00947-s001.zip › antibiotics-3562810-Supplementary.pdf]

SUPPLEMENTARY MATERIAL

Table S1. Primers and PCR conditions

Reaction	Name	Sequence (5'-3')	Target region	Amplicon size	Tm*	Isolate
1	GN628	GATGAAACGGCTGATTGCCC	*tnpA*-IS*Kpn6*	828	55°C	30Pae2
	GN716	CGAGACGCACTTTGTAGGCA	p30Pae2-KPC-Tn*4401b*			
	GN656	TTTGGTGCGTGTTGCGAAG	*tnpR*-Tn*4401b*	561		
	GN715	CCCACCGAATCCCAACCATC	p30Pae2-KPC-Tn*4401b*			
	GN717	GAACCACCGAAGAAGCCCAC	*trbG/virB9-F*	338		
	GN718	CTTTACCTCCCGCACCTCAC	*trbG/virB9-R*			
2	GN735	ATTCTGGGCGATTCTCGTGG	34Pae36-IS*Pa38*-1	633	61 °C	34Pae36
	GN736	CATGCCGGGCTGAACAAAG	*tnpA*-IS*Pa38*			
	GN737	CACCGACTCCATCGACACC	*tnpR*-IS*Pa38*	314		
	GN738	GATGACGGTAGGGGAACTGG	*ΔrepA*-34Pae36-1			
	GN739	CTGCTGCTTCAATGGGGAGA	*ΔrepA*-34Pae36-2	562		
	GN740	GATCTGCACGACCGCATCTT	*ΔtnpA*-34Pae36-2	768		
3	GN741	GCCTGAGCATTTAGCTGTGT	p30Pae23-Tn*2*	555	61 °C	34Pae23
	GN742	TCTGTCACGGTCAAAAAGGTG	*tnpA*-Tn*2*			
	GN743	AGCCCTCCCGTATCGTAGTT	*Δbla*$_{TEM-1}$	412		
	GN744	TCGCGACTAATGGTGGATGG	*tnpA*-IS*Kmi1*			
	GN745	GATTGGGTGACTGGGAGGTG	*tnpA*-IS*Kmi1*	689		
	GN746	CGGCCATGAGAGACAAGACA	*bla*$_{KPC-2}$			
	GN747	TCTGACACTGCCCAAGGTTC	*Δ*IS*Kpn16*-p34Pae23	286		

	GN749	CTTTGCCAGAACAGCCTCAC	*ΔtnpA*-IS*Kpn6*	805		

*PCR conditions: 95° C * 5´, [30 cycles (95° C *30", Tm*45", 72° C * 1], 72°C * 5’.

Table S2. Antimicrobial susceptibility and co-resistance profiles of the *Pseudomonas aeruginosa* isolates**.**

<table>
<tr><th>Co- re-sistance</th><th>ß-lactams</th><th>Aminoglycosides</th><th>Quinolones</th><th>Polymixin</th><th>n</th><th>%</th><th>n</th><th>%</th></tr>
<tr><td>1</td><td style="background:#ccc"></td><td></td><td></td><td></td><td>9</td><td>7.0</td><td>9</td><td>7.0</td></tr>
<tr><td rowspan="4">2</td><td style="background:#ccc"></td><td></td><td style="background:#ccc"></td><td></td><td>3</td><td>2.3</td><td rowspan="4">18</td><td rowspan="4">14.2</td></tr>
<tr><td style="background:#ccc"></td><td style="background:#ccc"></td><td></td><td></td><td>3</td><td>2.3</td></tr>
<tr><td style="background:#ccc"></td><td></td><td></td><td></td><td>3</td><td>2.3</td></tr>
<tr><td style="background:#ccc"></td><td></td><td></td><td style="background:#ccc"></td><td>9</td><td>7.0</td></tr>
<tr><td rowspan="2">3</td><td style="background:#ccc"></td><td style="background:#ccc"></td><td style="background:#ccc"></td><td></td><td>91</td><td>71.7</td><td rowspan="2">92</td><td rowspan="2">72.4</td></tr>
<tr><td style="background:#ccc"></td><td style="background:#ccc"></td><td></td><td style="background:#ccc"></td><td>1</td><td>0.8</td></tr>
<tr><td>4</td><td style="background:#ccc"></td><td style="background:#ccc"></td><td style="background:#ccc"></td><td style="background:#ccc"></td><td>8</td><td>6.3</td><td>8</td><td>6.3</td></tr>
<tr><td>Total*</td><td>127 (99,2%)</td><td>106 (84,1%)</td><td>102 (81,6%)</td><td>18 (16,5%)</td><td>127</td><td>99.2</td><td>127</td><td>99.2</td></tr>
</table>

***** The antimicrobial susceptibility information was not obtained for one isolate.

Table S3. Resistome of *Pseudomonas aeruginosa* isolates ST111 and ST235

Resistance phenotype	Resistance gene	30Pae2 (ST111)	34Pae8 (ST235)	34Pae23 (ST235)	34Pae36 (ST111)
Phenicol	*catB7*	Dark blue	Dark blue	Dark blue	Dark blue
Sulphonamide	*sul1*	Dark blue	Dark blue	Dark blue	Dark blue
	aac(3)-Iia	Dark blue	Dark blue	Dark blue	Dark blue
	aac(6')-29	Dark blue	Light blue	Light blue	Dark blue
	aac(6')-Ian	Dark blue			
Aminoglycoside	*aph(3'')-Ib*	Dark blue			
	aph(3')-IIb	Dark blue			
	aph(6')-Id	Dark blue			
	aadA6	Dark blue			
	aadA13	Dark blue			
Antiseptics	*qacEΔ1*	Dark blue			
Fosfomycin	*fosA*	Dark blue	Dark blue	Dark blue	Dark blue
	bla$_{PDC}$	Dark blue	Dark blue	Dark blue	Dark blue
	bla$_{OXA-488}$	Dark blue			Dark blue
	bla$_{OXA-395}$	Dark blue			Dark blue
β-lactams	*bla*$_{TEM}$	Dark blue			Dark blue
	bla$_{KPC-2}$		Light blue	Light blue	
	bla$_{KPC-3}$	Light blue			Dark blue
	bla$_{VIM-2}$	Dark blue			Dark blue

Dark blue: Chromosome localization.
Light blue: Plasmid localization

Table S4. Primary strategies that can be implemented in hospitals to reduce antimicrobial resistance (AMR):

Strategy	Description	Key Actions	Expected Impact	References
**1. Antimicrobial Stewardship Programs (ASPs)**	Systematic efforts to optimize antibiotic use, ensuring appropriate prescribing.	- Form multidisciplinary ASP team. - Develop hospital-specific guidelines. - Use prospective audit and feedback. - Implement pre- authorization for restricted antibiotics. - Promote short-course therapies.	Reduces inappropriat e use by 10-30%, lowers resistance rates.	[48 - 49]
**2. Enhance Diagnostic Precision**	Use rapid diagnostics and biomarkers to target therapy, minimizing broad- spectrum use.	- Deploy PCR, MALDI-TOF for pathogen ID. - Use procalcitonin to guide therapy. - Ensure high-quality susceptibility testing.	Reduces broad- spectrum use, limits MDRO selection.	[50 - 51]
**3. Infection Prevention and Control (IPC)**	Prevent infections to reduce antibiotic need.	- Enforce hand hygiene. - Use contact precautions for MDROs. - Enhance environmental cleaning. - Promote device stewardship.	Decreases HAIs, reducing antibiotic exposure (e.g., 50% CLABSI reduction).	[52, 53]
**Strategy**	**Description**	**Key Actions**	**Expected Impact**	**References**

Strategy	Description	Key Actions	Expected Impact	References
		- Vaccinate staff/patients.		
**4. Educate Healthcare Staff**	Train staff to improve prescribing practices and awareness of resistance.	- Conduct regular training. - Use case-based learning. - Disseminate protocol guides. - Engage leadership for accountability.	Reduces unnecessary prescriptions by 20-40%.	[54, 55]
**5. Promote De-escalation**	Switch to narrow- spectrum antibiotics or stop therapy when unnecessary.	- Review therapy at 48-72 hours. - Use evidence-based stop dates. - Avoid prolonged surgical prophylaxis.	Reduces broad-spectrum exposure, lowers resistance risk.	[56, 57]
**6. Limit Broad-Spectrum Antibiotics**	Restrict use of broad-spectrum and last-resort antibiotics to preserve efficacy.	- Restrict to confirmed resistant cases. - Rotate antibiotic classes in ICUs. - Use cycling/mixing strategies.	Preserves efficacy, reduces MDRO prevalence (e.g., lower MRSA rates).	[58-59]
**7. Monitor Antibiotic Use and Resistance**	Track consumption and resistance to guide interventions .	- Measure DDD/DOT metrics. - Update antibiograms regularly. - Join surveillance networks (e.g., GLASS). - Provide	Identifies high-use areas, reduces resistance by up to 15%.	[60-61]
		prescriber feedback.		

**8. Engage Patients and Families**	Educate patients to reduce demand for unnecessary antibiotics.	- Educate on resistance risks.<br>- Use decision aids/posters.<br>- Involve in shared decision-making.	Reduces unnecessary prescriptions by up to 25%.	[62-63)
**9. Address Environmental/Communit y Linkages**	Coordinate with community to reduce resistance spread.	- Standardize stewardship across facilities.<br>- Promote antibiotic disposal programs.<br>- Support community vaccination/IPC.	Lowers community-acquired resistance, reducing hospital burden.	[64]
